# Editorial: Pathways of risk, resilience, and recovery: impact of stress and trauma on women and girls

**DOI:** 10.3389/fpsyt.2023.1290535

**Published:** 2023-09-28

**Authors:** Liat Helpman, Dana Lassri, Rachel G. Zsido, Catherine Monk, Maria R. Dauvermann

**Affiliations:** ^1^Department of Counseling and Human Development, Faculty of Education, University of Haifa, Haifa, Israel; ^2^Paul Baerwald School of Social Work and Social Welfare, Hebrew University of Jerusalem, Jerusalem, Israel; ^3^Department of Psychiatry, Massachusetts General Hospital, Harvard Medical School, Boston, MA, United States; ^4^Max Planck Institute for Human Cognitive and Brain Sciences, Leipzig, Lower Saxony, Germany; ^5^New York State Psychiatric Institute (NYSPI), New York, NY, United States; ^6^Department of Obstetrics and Gynecology, Vagelos College of Physicians and Surgeons, Columbia University Irving Medical Center, New York, NY, United States; ^7^School of Psychology, Institute for Mental Health, University of Birmingham, Birmingham, United Kingdom

**Keywords:** sex, gender, trauma, stress, biopsychosocial (BPS) model

## Introduction

Stress and trauma are ubiquitous experiences that have been identified as transdiagnostic factors associated with a higher risk for disproportionately detrimental physical and mental health outcomes for women and girls, including posttraumatic and affective disorders (1, 2). The underlying mechanisms of this increased risk likely involve complex biopsychosocial processes that have yet to be fully identified (3). Furthermore, the role of protective and resilience factors buffering these associations remain relatively unexamined. In this Research Topic, we aim to address this complexity from various interdisciplinary perspectives and discuss the biological, psychological, and social factors that may underpin both risk and resilience in the face of stressful and traumatic experiences.

This collection of research includes biological substrates of risk, such as neural (Eder-Moreau et al.), genetic (Carvalho et al.) and endocrine (Brouillard et al.) factors. It also addresses potential social determinants of poor health, such as economic precarity and social isolation (Pazderka et al.) as well as the co-occurrence among mental health, risky behavior, and infectious disease among women released from incarceration (Johnson et al.). Social determinants also hold the potential for buffering potentially negative impact, through resources accessed in the face of adversity (Zamir et al.). The psychological underpinnings that may help explain the associations between stressful experience and compromised outcomes are also explored. These include interpretation of stressors from a social perspective (Azoulay and Gilboa-Schechtman) as well as from a psychological perspective, such as mentalizing (Ensink et al.). Finally, this Research Topic considers potential mechanisms for familial, intergenerational effects of maternal stress, such as parenting (Ahmad et al.).

## Biological substrates of stress-related disorders (SRDs) among women

A study investigating epigenetic changes in immune cells following trauma found that shortened leukocyte telomere length observed among women exposed to sexual assault was associated with re-experiencing post-trauma symptoms. However, this association between trauma and a marker of cellular aging did not persist over time, possibly indicating a temporary and reversible effect near the time of traumatic event (Carvalho et al.).

A systematic review of neural patterns among adult women exposed to trauma showed that the type of trauma (i.e., interpersonal violence, sexual trauma, and childhood trauma) is associated with specific neural regions. While the amygdala and frontal regions of the frontoparietal network were implicated in all trauma types, only childhood trauma was related to parietal regions of this network and with the hippocampus. Interpersonal violence was the only type of trauma consistently associated with the anterior cingulate cortex and medial prefrontal cortex and was also related to altered insula activity and structure, as was sexual trauma. The caudate was implicated only in sexual trauma (Eder-Moreau et al.).

Lastly, lifetime usage of hormonal contraceptives was identified as a risk factor for psychological distress during stressful experiences as it was associated with poor mental health during the COVID-19 pandemic: it was associated with both more severe and persistent symptoms (Brouillard et al.).

Taken together, these articles suggest that there are specific and enduring brain structural and functional alterations in adult women that are associated with specific timings and types of traumatic exposures, and that epigenetic changes may occur, albeit temporarily. Also, the utilization of oral contraceptives may be associated with risk for maladjustment following stress, thereby underscoring the potential role of gonadal hormones in stress response and subsequent adjustment.

## Social determinants of SRDs among women

Social determinants of health encompass social variables that are associated with the risk of disease (4), its severity (5), access to care (6), and the recovery process (7). Such variables include social support, socioeconomic status, community factors, race, ethnicity among others (4, 8, 9). Social determinants of health have also been associated with SRDs. Within this collection, several studies have examined risk and resilience factors for women in high-risk, underserved populations.

In a survey of women living through the COVID-19 pandemic in a remote town considered to be inhospitable to women due to living in temporary accommodations, high crime rates, and a patriarchal reputation, the authors examined the impact of job loss, relationship status, access to mental health counseling or medication, and social support on probable diagnosis of PTSD (Pazderka et al.). Under these particularly harsh conditions, support from family and friends as well as negative stressors of job loss, but not access to mental health treatment or relationship status, predicted posttraumatic stress.

Women who have been released from incarceration constitute a vulnerable population as they have frequently been exposed to trauma prior to and during incarceration (Lynch and Heath (10) found that this marginalized group is particularly vulnerable to both SRDs and infections, in particular, sexually transmitted infections, including human immunodeficiency virus and hepatitis C (11, 12). As SRDs are associated with low adherence to healthcare treatment, increased infection risk is of particular concern. The WORTH Transitions is an intervention developed to reduce risk of additional trauma, prevent infection, and promote healthy behaviors among these high-risk women in a culturally appropriate treatment. The study of this intervention found that PTSD was negatively associated with session engagement and positively correlated with loss to follow up, but not with risk of infection. Being a black or indigenous woman of color was also associated with lower engagement, underscoring the need to tailor interventions to better meet the need of this marginalized population.

An additional study examined the association between stressors during pregnancy and mother and child's outcomes postpartum (Ahmad et al.). The study highlights the vulnerability of women from underserved communities of color with lower incomes who face a higher risk of experiencing prenatal stressors.

Social determinants of health have an impact on the general population as well. In one article (Zamir et al.) authors examine such impact for women contending with breast cancer a women with breast cancer. This study highlights the impact of family income level and partner support in parenting on maternal post-traumatic symptoms and parenting behaviors. Their findings suggest that partner support fully mediated the effects of income levels on maternal posttraumatic symptoms.

In summary, these studies underscore the importance of social determinants of health across several levels. On the community level, belonging to specific communities, such as minority or low socioeconomic status groups, constitutes a risk factor for traumatic exposure, as well as for low engagement with treatment programs, and requires community-level prevention and adherence support. On the interpersonal level, women benefit from the support of partners, family, and friends, which helps mitigate the effects of stressful and traumatic experiences, however the mere existence of partners appears not to be enough. Finally, economic disadvantage is a risk factor for the detrimental impact of stress and trauma, but its effect may also be explained, in some cases, by the availability of social support.

## Psychological underpinnings of SRDs among women

The systematic review included in this Research Topic showed that females with PTSD demonstrate greater emotional dysregulation than controls, as expressed both on the behavioral and neural level. Authors suggest that the findings reflect reduced neural activation when faced with positive stimuli and increased activation when faced with negative stimuli, related to the negative attention bias found in posttraumatic stress disorder. Authors further present data to support the relationship between these biases and traits like neuroticism as well as coping mechanisms, such as rumination. Women may, perhaps due to heightened neuroticism, be biased toward negative information, ruminate on this information, experience emotional dysregulation due to reductions in top-down control, expressed at the neural level, thus displaying a distinct symptom profile (Eder-Moreau et al.).

In one study, authors explored the associations between attachment, mentalizing, and posttraumatic stress symptoms among pregnant women with a history of childhood maltreatment. The authors present findings supporting mentalizing as a resilience factor and a potential mechanism for reducing symptoms within this population by mitigating the association between childhood maltreatment and SRDs (Ensink et al.).

Another study (Azoulay and Gilboa-Schechtman) suggests exploring sex difference in SRD prevalence through the lens of two theoretical frameworks: social construction theory vs. evolutionary theory. Social construction theory suggests women's increased risk for such disorders may be tied to lower perceived and actual social status, while the evolutionary theory suggests it depends on the interruption of specific sociobiological goals, and that women are more susceptible to physical threats, while men are more sensitive to status losses. An experimental design produced results consistent with the evolutionary theory such that status losses were not associated with posttraumatic distress among women.

Articles in this section suggest that women may be susceptible to SRDs due to gender biases in psychological traits, such as neuroticism, use of coping mechanisms, such as rumination, and negativity biases. However, women may also be protected from these effects by utilizing more adaptive coping mechanisms such as mentalization and may not be susceptible to the contributing effects of specific types of stressful events, such as status loss, to SRD development.

## SRDs and intergenerational (familial) effects

For women, SRDs are most prevalent during childbearing years (13). As a consequence, research on intergenerational effects of trauma has largely focused on mothers, as have several studies in this special section.

One study focused on mothers with breast cancer. These mothers experience compound stress, coping with the intensive treatments and their physical side effects as well as continued childcare demands. According to the family stress model, stressful conditions may cause emotional distress which may deplete the psychological resources of the parents, lending to harsh parenting practices. In this study, the authors found a positive association between maternal post-traumatic stress symptoms during breast cancer treatment and harsh parenting practices, and maternal posttraumatic stress symptoms fully mediated the association between paternal support and parenting practices (Zamir et al.).

However, motherhood can be conceptualized as beginning in the peripartum period. Stress experienced by mothers in the prenatal period affects their children's mental health and development. In the systematic review included here, findings suggest maternal prenatal stress associates with self-regulation problems, difficulties with executive functioning, and subsequent externalizing behavioral problems in children (Eder-Moreau et al.).

An intergenerational study examined this association between maternal stress and child outcomes within a diverse sample of mother-child dyads (Ahmad et al.). Their results emphasize the role of parenting as a protective factor and the importance of positive parent-child interactions and supportive parenting behaviors in mitigating intergenerational risks. Notably, intimate partner violence emerges as a prenatal stressor significantly associated with a child's subsequent executive functioning.

In the study discussed in the previous section (Ensink et al.), mentalizing regarding early attachment relationships moderated the association between childhood maltreatment and SRDs, specifically posttraumatic symptoms, among pregnant women. This finding, coupled with the study demonstrating the importance of maternal symptoms in determining parenting practices, suggests that the capacity to mentalize may serve as a potential mechanism for curbing intergenerational effects of trauma.

## Conclusions

Reviewing results across these studies, the interdisciplinary articles in this Research Topic examine the impact of multiple types of stressful experiences on the mental and physical health of women and girls: sexual assault, interpersonal stress, childhood maltreatment, breast cancer, and the COVID-19 pandemic. The findings suggest that sex- and gender-specific risk factors may include factors such as hormonal contraceptive use or the type of stressor experienced and its subjective perception. They also delineate potential protective and resilience factors that may mitigate the negative impact of stress and trauma on mental health outcomes. External interpersonal resources (such as familial, social, and partner support, parenting practices) as well as personal resources (such as the capacity to mentalize regarding parental relationships) and financial resources are all identified as potential buffers against adverse mental health effects. Taken together, the articles in this collection suggest that behavioral, neural, and endocrine mechanisms may underlie these processes. Better understanding of these mechanisms and their interactions will lead to more effective, targeted assessment and intervention practices for women and girls across development. Such practices would consider sex as a biological variable in risk assessment and intervention timing, as well as in pharmacologic and device-oriented intervention, and bear in mind structural, social, and familial factors that impact women specifically alongside individual, psychological factors. We hope the current collection will contribute to the growing research base that recognizes sex as a biological variable and gender as a psychosocial variable in the explanation of risk, recovery, and resilience in the face of adversity. We encourage the translation of such knowledge into sex-and gender-specific mental health practices. The findings described here may inform both intervention and policy by identifying specific protective factors that may be enhanced and risk factors that may be reduced.

## Author contributions

LH: Conceptualization, Project administration, Writing—original draft, Writing—review and editing. DL: Writing—review and editing. RZ: Writing—review and editing. CM: Writing—review and editing. MD: Writing—review and editing.
